# Biochemical and crystallization analysis of the CENP-SX–DNA complex

**DOI:** 10.1107/S2053230X22003843

**Published:** 2022-04-22

**Authors:** Sho Ito, Tatsuya Nishino

**Affiliations:** a Department of Biological Science and Technology, Faculty of Advanced Engineering, Tokyo University of Science (TUS), 6-3-1 Niijyuku, Katsushika-ku, Tokyo 125-8585, Japan

**Keywords:** protein–DNA interactions, histone-fold complexes, DNA repair, chromosome segregation, crystallization, CENP-SX–DNA complex

## Abstract

The CENP-SX (MHF) complex is a conserved histone-fold protein complex that is involved in chromosome segregation and DNA repair. It can bind to DNA on its own as well as in complex with other proteins such as CENP-TW and FANCM to recognize specific substrates. CENP-SX binds nonspecifically to dsDNA, similar to other histone-fold proteins. Several low-resolution structures of CENP-SX in complex with DNA are known, but a high-resolution structure is still lacking. The DNA-binding properties of CENP-SX and FANCM–CENP-SX complexes with various lengths of dsDNA were compared and the band-shift patterns and migration positions were found to differ. To confirm the DNA-binding properties in detail, CENP-SX–DNA and FANCM–CENP-SX–DNA complexes were crystallized. Analysis of the crystals revealed that they all contained the CENP-SX–DNA complex, irrespective of the complex that was used in crystallization. Detailed diffraction data analyses revealed that there were two types of crystal with different space groups, *P*2_1_ and *C*2, where the volume of the *P*2_1_ asymmetric unit is twice as large as that of the *C*2 asymmetric unit. Analysis of the self-rotation function revealed the presence of twofold and fourfold symmetry in both crystals. This suggests that there may be multiple molecules of CENP-SX and DNA within the asymmetric unit with respective symmetry. Structure determination of the present crystals should reveal details of the DNA-binding properties of CENP-SX.

## Abstract 2

CENP-SX is a histone-fold complex that is involved in chromosome segregation and DNA repair. Biochemical and crystallization analysis suggested that multiple molecules of CENP-SX may be involved in DNA binding.

## 1. Introduction

Genome integrity is of the utmost importance in all living organisms. Eukaryotes, in particular, undergo mitotic and meiotic cell cycles to proliferate and produce the next generation. Chromosome segregation and DNA repair play pivotal roles in these processes. The CENP-S (MHF1)–CENP-X (MHF2) (CENP-SX) complex (also known as the MHF complex) is a conserved histone-fold complex that participates in these processes (Milletti *et al.*, 2020 ▸; Kixmoeller *et al.*, 2020 ▸). In chromosome segregation, it forms a complex with another kinetochore component, CENP-T–CENP-W, to form a heterotetrameric CENP-TWSX complex (Nishino *et al.*, 2012 ▸). As part of the kinetochore machinery, it connects chromosome and spindle microtubules during mitosis (Kixmoeller *et al.*, 2020 ▸). In the absence of the CENP-SX complex, the kinetochore structure becomes abnormal and mis-segregation becomes prominent (Amano *et al.*, 2009 ▸). During DNA repair, the CENP-SX complex interacts with FANCM to form the FANCM–CENP-SX complex (Singh *et al.*, 2010 ▸; Yan *et al.*, 2010 ▸). FANCM is a core component of the Fanconi anemia (FA) pathway and plays a vital role in the localization of the other proteins to the DNA damage site (Milletti *et al.*, 2020 ▸).

Biochemical analysis of the purified CENP-SX complex has revealed that it forms a heterotetramer similar to histone H3/H4 (Nishino *et al.*, 2012 ▸; Tao *et al.*, 2012 ▸). It binds to dsDNA using the histone fold and the basic tail at the C-terminus of CENP-S (Nishino *et al.*, 2012 ▸). Interestingly, the binding pattern of the CENP-SX complex to DNA revealed that it forms a regularly spaced protein–DNA complex and the number of proteins increases with the DNA length. Intriguingly, addition of CENP-TW to the CENP-SX–DNA mixture leads to a loss of these regular binding patterns and the CENP-TWSX complex prefers to bind to ∼100 bp dsDNA. Purified human FANCM–CENP-SX complex has been shown to prefer to bind to branched molecules over dsDNA (Tao *et al.*, 2012 ▸; Fox *et al.*, 2014 ▸). The low-resolution crystal structure of human CENP-SX in complex with 26 bp dsDNA revealed that CENP-SX uses both its histone-fold and C-terminal basic tail regions in binding to dsDNA (Zhao *et al.*, 2014 ▸). Each CENP-SX dimer was bound to a separate dsDNA duplex and the overall shape resembled a branched DNA molecule. However, the mechanism of regularly spaced DNA binding and branch DNA binding by CENP-SX remains elusive.

Here, using chicken CENP-SX and FANCM–CENP-SX complexes, we tried to perform high-resolution structure analysis of their complexes with DNA. We obtained several crystals of CENP-SX–DNA using different lengths of dsDNA, some of which diffracted to ∼3.2 Å resolution. These crystals could be separated into two different space groups, each containing multiple molecules in the asymmetric unit. The space group and unit-cell parameters differ from those of the reported complex crystal structures. Thus, determination of the crystal structure should reveal details of the recognition mode. Phase determination and further refinement of the CENP-SX–DNA structure are currently in progress.

## 2. Materials and methods

### 2.1. Macromolecule production and electrophoretic mobility shift assay (EMSA)

FANCM–CENP-SX was prepared according to a previous study, replacing truncated CENP-S with full-length CENP-S (Ito & Nishino, 2021 ▸; Table 1 ▸). CENP-SX was prepared according to a previous report (Nishino *et al.*, 2012 ▸). Synthetic oligonucleotides based on the Widom 601 sequence were purchased from Thermo Fisher. Double-stranded DNAs (dsDNAs) were prepared by heat-annealing the complementary oligonucleotides. The dsDNAs were further purified by size-exclusion chromatography in 10 m*M* Tris pH 7.5, 100 m*M* NaCl.

EMSA was performed as described previously (Nishino *et al.*, 2012 ▸). Briefly, the CENP-SX tetramer or the FANCM–CENP-SX pentamer (1.25 µ*M*) was incubated with dsDNA of various lengths (1.25 µ*M*) at 42°C for 60 min in binding buffer (10 m*M* Tris–HCl pH 7.5, 100 m*M* NaCl). The mixtures were analyzed by 10–20% gradient native PAGE (Wako) and stained with ethidium bromide.

### 2.2. Crystallization

To form a protein–DNA complex, a mixture of protein and DNA was incubated at 20°C for 60 min. Initial crystallization screenings for FANCM–CENP-SX–dsDNA were performed using Natrix and Natrix 2 (Hampton Research) by the sitting-drop vapor-diffusion technique in a 96-well format crystallization plate. The final volume of the drop was 0.2 µl, with 0.1 µl of the reservoir solution and the protein–DNA complex, and the plate was incubated at a constant temperature of 20°C. Initial crystals were obtained in two conditions: Natrix condition No. 12 (25% MPD, 20 m*M* MgSO_4_, 50 m*M* cacodyl­ate pH 6.0) using 31 bp DNA and Natrix 2 condition No. 29 (30% 1,4-dioxane, 10 m*M* MgCl_2_, 2 m*M* NaCl_2_, 50 m*M* MOPS pH 7.0) using 19–49 bp DNA. For diffraction analysis, 1,4-dioxane was replaced by 30% MPD and the crystals were cryoprotected using 30% ethylene glycol.

CENP-SX–dsDNA crystallization was performed using 29–31 bp DNA. To improve the crystallization, the mixing ratio of protein and DNA, the DNA length and the overhang structures were varied. The optimized crystal condition was 20 m*M* MES–NaOH pH 6.5, 40% MPD, 100 m*M* NaCl with cryoprotection using 20% ethylene glycol, 15% dimethyl sulfoxide (DMSO).

Conditions for the production of CENP-SX–dsDNA crystals with improved diffraction quality are summarized in Table 2 ▸.

### 2.3. Data collection and processing

Diffraction data were collected on BL-1A at the Photon Factory (PF) synchrotron facility (KEK) and were processed with the *HKL*-2000 package (HKL Research) or *XDS* (Kabsch, 2010 ▸). Data analyses were performed using *MOLREP* from the *CCP*4 suite (Winn *et al.*, 2011 ▸). Data-collection and processing statistics are summarized in Table 3 ▸.

## 3. Results and discussion

**Figure 1:**
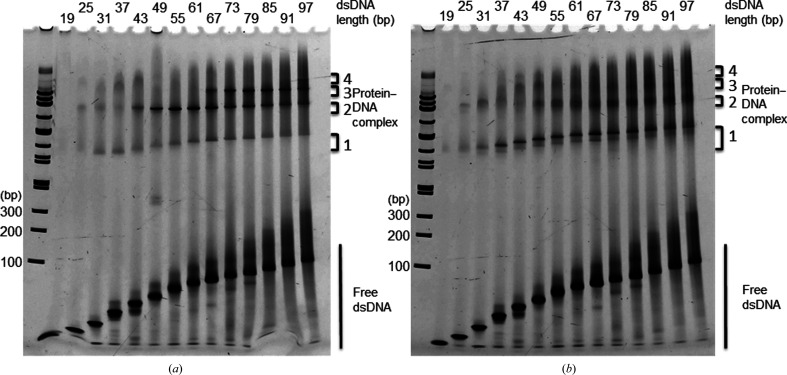
DNA-binding modes of (*a*) the CENP-SX tetramer and (*b*) the FANCM–CENP-SX pentamer to dsDNA of various lengths. (*a*) EMSA of CENP-SX (1.25 µ*M*) with 19, 25, 31, 37, 43, 49, 55, 61, 67, 73, 79, 85, 91 and 97 bp dsDNA (1.25 µ*M*). (*b*) EMSA of FANCM–CENP-SX (1.25 µ*M*) with the same set of dsDNAs (1.25 µ*M*) as used in (*a*). Protein–DNA complex bands are numbered accordingly.

Chicken and human CENP-SX bind a single dsDNA at regular intervals, whereas human FANCM–CENP-SX prefers branched molecules (Nishino *et al.*, 2012 ▸; Fox *et al.*, 2014 ▸; Zhao *et al.*, 2014 ▸). To compare the binding patterns in more detail, we performed EMSA with chicken CENP-SX and FANCM–CENP-SX using synthetic dsDNAs of various lengths (19–97 bp) based on the Widom 601 sequence. Consistent with the previous report, the number of CENP-SX–DNA bands increased with DNA length (Fig. 1 ▸; Nishino *et al.*, 2012 ▸). There were four shifted bands [1 (fast migration band) to 4 (slow migration band)] which contained CENP-SX or FANCM–CENP-SX as confirmed by Coomassie staining. Band 1 of the CENP-SX–DNA complex was absent for 19 bp dsDNA and a faint band started to appear for dsDNA of 25 bp up to 97 bp. Band 2 also started to appear from 25 bp DdsNA and its intensity increased dramatically from 49 bp dsDNA and peaked at 61 bp dsDNA. Interestingly, the intensity of band 2 was stronger and sharper than that of band 1. Band 3 was similar to band 2 and started to appear from 67 bp dsDNA. Band 4 only appeared for 97 bp dsDNA. FANCM–CENP-SX–DNA bands appeared similarly; however, the intensity of band 1 was stronger and sharper than that for CENP-SX–DNA. The other bands were less intense and were smeared. These results suggest that the DNA-binding mode and stoichiometry of CENP-SX differ in the presence and absence of FANCM.

**Figure 2:**
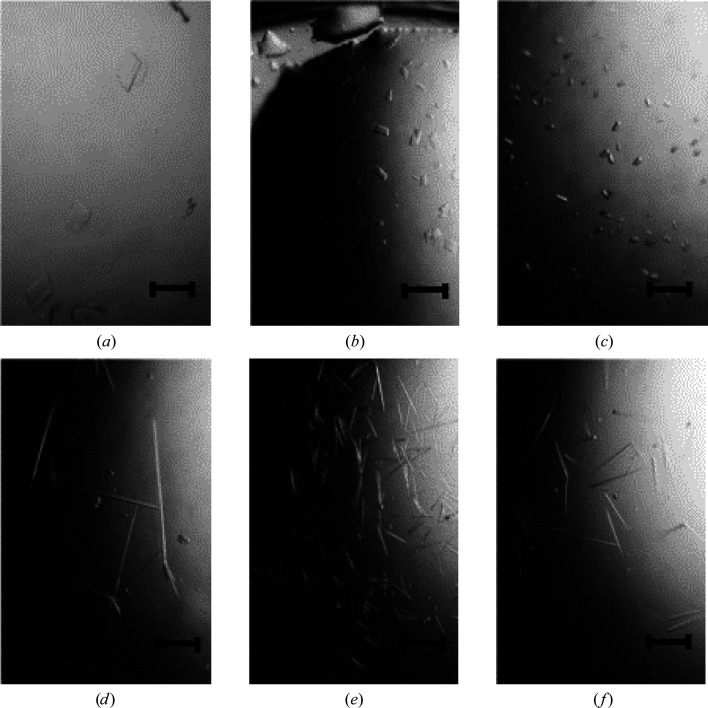
Crystals of the FANCM–CENP-SX–DNA complex obtained using various lengths of dsDNA. (*a*) 19 bp, (*b*) 25 bp, (*c*) 31 bp, (*d*) 37 bp, (*e*) 43 bp, (*f*) 49 bp. The scale bar is 0.2 mm in length.

**Figure 3:**
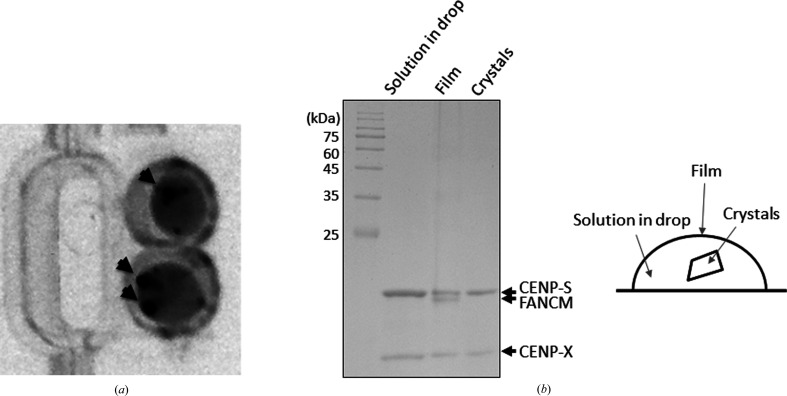
Analysis of FANCM–CENP-SX–DNA complex crystals. (*a*) Analysis of the crystals with fluorescent DNA-staining dye. The fluorescent image is shown in grayscale. The arrowheads indicate stained crystals. (*b*) Left: analysis of each component by 15% SDS–PAGE. The gel was stained with Coomassie Brilliant Blue. Right: schematic drawing of the sitting-drop crystallization setup indicating the crystals, solution and film.

To delineate the difference in DNA binding between the two complexes, we performed crystallization experiments in the presence of dsDNA (19, 25, 31, 37, 43 and 49 bp). Irrespective of the length of DNA used, FANCM–CENP-SX–DNA crystals appeared in the presence of 30% 1,4-dioxane. The shapes of the crystals differed according to the length of the DNA (Fig. 2 ▸). Rectangular crystals were formed using 19, 25 and 31 bp dsDNA. Needle-shaped crystals appeared using 37, 43 and 49 bp dsDNA. The contents of the crystals were analyzed by two different methods. Addition of DNA-staining green fluorescent dye to the crystal drop resulted in crystals that glowed green (Fig. 3 ▸ *a*). Analysis by SDS–PAGE revealed that the crystals contained CENP-S and CENP-X, whereas FANCM was absent (Fig. 3 ▸ *b*). FANCM was present as a film-like structure in the air–liquid interface of the crystal droplet. This situation is similar to a previous report where FANCM was observed to detach from CENP-SX in the presence of organic solvent and oxidative conditions (Ito & Nishino, 2021 ▸). Thus, FANCM detached from CENP-SX during crystallization even in the presence of DNA. Attempts to reproduce the CENP-SX–DNA crystal using a mixture of CENP-SX and DNA with the same precipitant were unsuccessful and the crystallization conditions were optimized. CENP-SX–DNA crystals appeared in the presence of 40% MPD.

**Figure 4:**
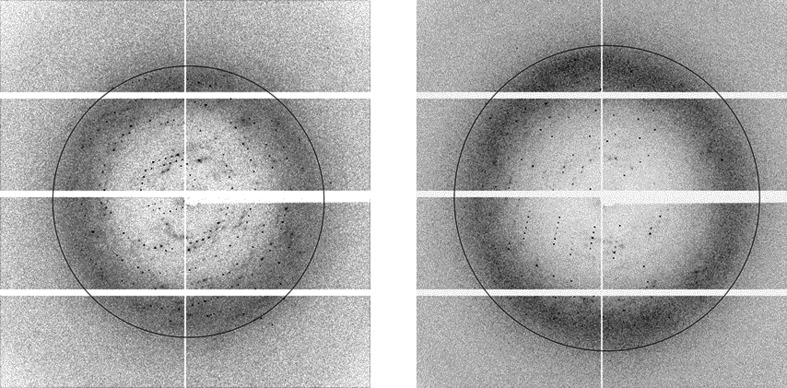
X-ray diffraction images of *P*2_1_ (left) and *C*2 (right) CENP-SX–DNA complex crystals collected on BL-1A at the Photon Factory, Japan. The circles indicate 3 Å resolution.

The initial crystals diffracted to ∼7 Å resolution with high mosaicity. Optimization of the DNA and cryoprotectant improved the resolution (Fig. 4 ▸). Data analysis showed that there were two different crystals with different space groups and unit-cell parameters. These crystals are both rectangular and are indistinguishable based on their shape. One crystal belonged to space group *P*2_1_, with unit-cell parameters *a* = 101, *b* = 84, *c* = 112 Å, α = 90, β = 105, γ = 90°. The other crystal belonged to space group *C*2, with unit-cell parameters *a* = 128, *b* = 81, *c* = 100 Å, α = 90, β = 124, γ = 90° (Table 3 ▸). The volume of the asymmetric units of the two crystals differs by twofold. Matthews analysis of the two crystals indicated that the asymmetric units of the *C*2 and *P*2_1_ crystals contain ∼80 000 and ∼160 000 Da, respectively, with a calculated Matthews coefficient of 2.7 Å^3^ Da^−1^ and a solvent content of 60%. These results suggest that multiple CENP-SX heterodimers and DNA are present in the asymmetric unit. The situation resembles previous low-resolution CENP-SX–DNA complex crystal structures, in which several different crystals were formed and multiple molecules were present in the asymmetric units.

**Figure 5:**
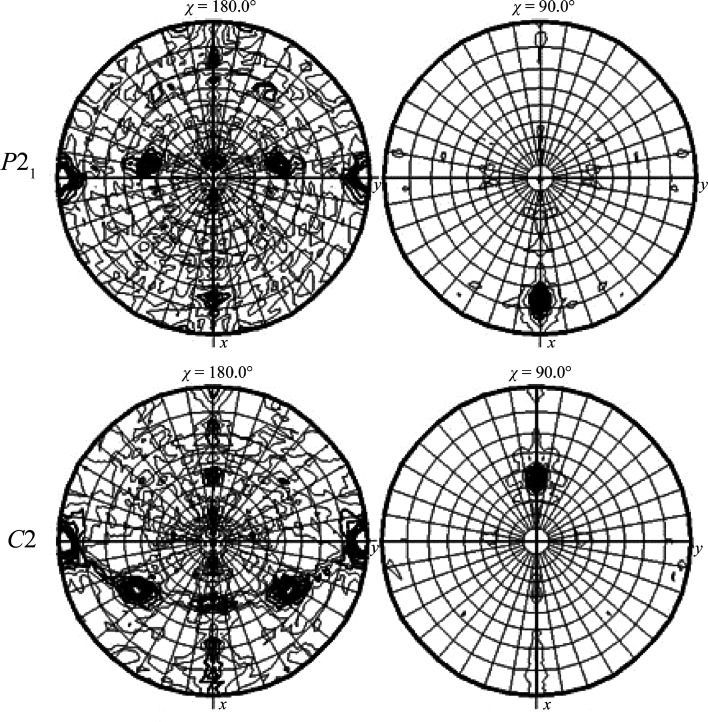
Self-rotation functions of the *P*2_1_ (top) and *C*2 (bottom) CENP-SX–DNA crystals. *MOLREP* was used for calculations. Inspection of the χ = 180° and 90° sections indicate the presence of twofold and fourfold symmetry in both crystals.

To analyze the relationship between the multiple molecules of CENP-SX and DNA within the crystal, the self-rotation function was calculated. In both the *P*2_1_ and *C*2 crystals, twofold peaks in the *ac* plane and fourfold peaks in the 90° plane (Fig. 5 ▸) were observed. The twofold symmetry may be due to the symmetry of the CENP-SX tetramer. Alternatively, there may be a twofold-symmetric CENP-SX–DNA complex similar to the reported complex structure, in which CENP-SX dimer–dsDNA complexes were related by twofold symmetry (Zhao *et al.*, 2014 ▸). However, an explanation of the fourfold peak remains elusive. EMSA analyses revealed that multiple CENP-SX tetramers bind to a discrete length of dsDNA. Structure determination should reveal the details of the recognition mechanism. Model building and structure refinement are currently in progress.

## Figures and Tables

**Table 1:** Macromolecule-production information The introduction of additional residues, the expression and purification tags, and TEV recognition sites are underlined. Cleavage sites are indicated with a slash.

	FANCM–CENP-SX	CENP-SX
Source organism	*Gallus gallus*	*Gallus gallus*
DNA source	cDNA	cDNA
Cloning vector	pMal-c2X	pRSFDuet-1
Expression vector	pMal-c2X	pRSFDuet-1
Expression host	*E. coli* One Shot BL21(DE3) pLysS RARE2	*E. coli* One Shot BL21(DE3) pLysS RARE2
Complete amino-acid sequence of the construct produced		
CENP-S	MGSHHHHHHENLYFQ/GSEAAGGEQRELLIQRLRAAVHYTTGALAAQDVAEDKGVLFSKQTVAAISEITFRQAENFARDLEMFARHAKRSTITSEDVKLLARRSNSLLKYITQKSDELASSNMEQKEKKKKKSSAAKGRKTEENETPVTESEDSNMAT	MGSHHHHHHENLYFQ/GSEAAGGEQRELLIQRLRAAVHYTTGALAAQDVAEDKGVLFSKQTVAAISEITFRQAENFARDLEMFARHAKRSTITSEDVKLLARRSNSLLKYITQKSDELASSNMEQKEKKKKKSSAAKGRKTEENETPVTESEDSNMAT
CENP-X	MYWSHPQFEKENLYFQ/GYEEREGGFRKETVERLLRLHFRDGRTRVNGDALLLMAELLKVFVREAAARAARQAQAEDLEKVDIEHVEKVLPQLLLDFV	MYWSHPQFEKENLYFQ/GYEEREGGFRKETVERLLRLHFRDGRTRVNGDALLLMAELLKVFVREAAARAARQAQAEDLEKVDIEHVEKVLPQLLLDFV
FANCM	MKIEEGKLVIWINGDKGYNGLAEVGKKFEKDTGIKVTVEHPDKLEEKFPQVAATGDGPDIIFWAHDRFGGYAQSGLLAEITPDKAFQDKLYPFTWDAVRYNGKLIAYPIAVEALSLIYNKDLLPNPPKTWEEIPALDKELKAKGKSALMFNLQEPYFTWPLIAADGGYAFKYENGKYDIKDVGVDNAGAKAGLTFLVDLIKNKHMNADTDYSIAEAAFNKGETAMTINGPWAWSNIDTSKVNYGVTVLPTFKGQPSKPFVGVLSAGINAASPNKELAKEFLENYLLTDEGLEAVNKDKPLGAVALKSYEEELAKDPRIAATMENAQKGEIMPNIPQMSAFWYAVRTAVINAASGRQTVDEALKDAQTNSSSNNNNNNNNNNLGIEGRHHHHHHENLYFQ/GRENLYFQ/GENLYFQ/GRSLHHKSALFSCVTDPKEMHCHENWSLSPEEFEIWDRLYRLKENDGVKEPILPHTRFETLENLDKTSKPEEEAAHKLSLSEWSIWQSRPFPTSMVDHSDRCYHFISVMELIEVMRQEQGDCSYELELQPHLRIEDIHVRRNKGHLSP	

**Table 2:** Crystallization

Method	Vapor diffusion, sitting drop	Vapor diffusion, sitting drop
Plate type	CrystalQuick Greiner plate, 3-well, round	CrystalQuick Greiner plate, 3-well, round
Temperature (K)	293	293
Protein concentration (µ*M*)	50 (FANCM–CENP-SX pentamer)	150 (CENP-SX tetramer)
DNA concentration (µ*M*)	55	55
Buffer composition of protein–DNA solution	10 m*M* MOPS–NaOH pH 7.0, 110 m*M* NaCl, 0.5 m*M* DTT	10 m*M* Tris–HCl pH 7.5, 110 m*M* NaCl
Composition of reservoir solution	50 m*M* MOPS–NaOH pH 7.0, 31% 1,4-dioxane, 10 m*M* MgCl_2_, 110 m*M* NaCl	20 m*M* MES–NaOH pH 6.5, 40% MPD, 100 m*M* NaCl
Volume of reservoir (µl)	100	100
Volume and ratio of drop	2 µl (1:1)	2 µl (1:1)
Composition of harvesting solution	50 m*M* MOPS–NaOH pH 7.0, 40% MPD, 10 m*M* MgCl_2_, 110 m*M* NaCl	—
Cryoprotectant	50 m*M* MOPS–NaOH pH 7.0, 30% MPD, 10 m*M* MgCl_2_, 110 m*M* NaCl, 30% ethylene glycol	20 m*M* MES–NaOH pH 6.5, 40% MPD, 100 m*M* NaCl, 20% ethylene glycol, 15% DMSO

**Table 3:** Data collection and processing for CENP-SX–dsDNA Values in parentheses are for the outer shell.

Diffraction source	BL-1A, PF	BL-1A, PF
Wavelength (Å)	1.1	1.1
Detector	EIGER	EIGER
Space group	*P*2_1_	*C*2
*a*, *b*, *c* (Å)	100, 81.6, 110	127, 81.5, 100
α, β, γ (°)	90, 106, 90	90, 124, 90
Mosaicity (°)	0.095	0.095
Resolution range (Å)	44–3.6 (3.7–3.6)	44–3.2 (3.3–3.2)
Total No. of reflections	69125 (7187)	49154 (5154)
No. of unique reflections	20200 (2012)	14264 (1425)
Completeness (%)	98.7 (99.5)	99.3 (99.4)
Multiplicity	3.4 (3.6)	3.4 (3.6)
〈*I*/σ(*I*)〉	13.0 (4.7)	24.6 (7.2)
*R*_merge_	0.054 (0.23)	0.031 (0.16)
*R*_meas_	0.065 (0.28)	0.038 (0.19)
*R*_p.i.m._	0.035 (0.15)	0.020 (0.10)
Overall *B* factor from Wilson plot (Å^2^)	110.9	99.5
CC_1/2_	0.99 (0.96)	0.99 (0.98)
CC*	1 (0.99)	1 (0.99)

## References

[bb1] Amano, M., Suzuki, A., Hori, T., Backer, C., Okawa, K., Cheeseman, I. M. & Fukagawa, T. (2009). *J. Cell Biol.* **186**, 173–182. 10.1083/jcb.200903100 PMC2717651 19620631

[bb2] Fox, D., Yan, Z., Ling, C., Zhao, Y., Lee, D., Fukagawa, T., Yang, W. & Wang, W. (2014). *Cell Res.* **24**, 560–575. 10.1038/cr.2014.42 PMC4011343 24699063

[bb3] Ito, S. & Nishino, T. (2021). *Acta Cryst.* F**77**, 1–7. 10.1107/S2053230X20016003 PMC7805551 33439149

[bb4] Kabsch, W. (2010). *Acta Cryst.* D**66**, 125–132. 10.1107/S0907444909047337 PMC2815665 20124692

[bb5] Kixmoeller, K., Allu, P. K. & Black, B. E. (2020). *Open Biol.* **10**, 200051. 10.1098/rsob.200051 PMC7333888 32516549

[bb6] Milletti, G., Strocchio, L., Pagliara, D., Girardi, K., Carta, R., Mastronuzzi, A., Locatelli, F. & Nazio, F. (2020). *Cancers*, **12**, 2684. 10.3390/cancers12092684 PMC7565043 32962238

[bb7] Nishino, T., Takeuchi, K., Gascoigne, K. E., Suzuki, A., Hori, T., Oyama, T., Morikawa, K., Cheeseman, I. M. & Fukagawa, T. (2012). *Cell*, **148**, 487–501. 10.1016/j.cell.2011.11.061 PMC3277711 22304917

[bb8] Singh, T. R., Saro, D., Ali, A. M., Zheng, X.-F., Du, C., Killen, M. W., Sachpatzidis, A., Wahengbam, K., Pierce, A. J., Xiong, Y., Sung, P. & Meetei, A. R. (2010). *Mol. Cell*, **37**, 879–886. 10.1016/j.molcel.2010.01.036 PMC2848122 20347429

[bb9] Tao, Y., Jin, C., Li, X., Qi, S., Chu, L., Niu, L., Yao, X. & Teng, M. (2012). *Nat. Commun.* **3**, 782. 10.1038/ncomms1779 PMC3646547 22510687

[bb10] Winn, M. D., Ballard, C. C., Cowtan, K. D., Dodson, E. J., Emsley, P., Evans, P. R., Keegan, R. M., Krissinel, E. B., Leslie, A. G. W., McCoy, A., McNicholas, S. J., Murshudov, G. N., Pannu, N. S., Potterton, E. A., Powell, H. R., Read, R. J., Vagin, A. & Wilson, K. S. (2011). *Acta Cryst.* D**67**, 235–242. 10.1107/S0907444910045749 PMC3069738 21460441

[bb11] Yan, Z., Delannoy, M., Ling, C., Daee, D., Osman, F., Muniandy, P. A., Shen, X., Oostra, A. B., Du, H., Steltenpool, J., Lin, T., Schuster, B., Décaillet, C., Stasiak, A., Stasiak, A. Z., Stone, S., Hoatlin, M. E., Schindler, D., Woodcock, C. L., Joenje, H., Sen, R., de Winter, J. P., Li, L., Seidman, M. M., Whitby, M. C., Myung, K., Constantinou, A. & Wang, W. (2010). *Mol. Cell*, **37**, 865–878. 10.1016/j.molcel.2010.01.039 PMC2847587 20347428

[bb12] Zhao, Q., Saro, D., Sachpatzidis, A., Singh, T. R., Schlingman, D., Zheng, X.-F., Mack, A., Tsai, M.-S., Mochrie, S., Regan, L., Meetei, A. R., Sung, P. & Xiong, Y. (2014). *Nat. Commun.* **5**, 2987. 10.1038/ncomms3987 PMC3967914 24390579

